# An investigation of simple neural network models using smartphone signals for recognition of manual industrial tasks

**DOI:** 10.1038/s41598-025-06726-y

**Published:** 2025-07-11

**Authors:** Tacjana Niksa‑Rynkiewicz, Panorios Benardos, Anna Witkowska, George-Christopher Vosniakos

**Affiliations:** 1 Faculty of Ocean Engineering and Ship Technology, Institute of Manufacturing and Materials Technology, Gdańsk University of Technology, 11/12 Gabriela Narutowicza Street, 80‑233, Gdańsk, Poland; 2 School of Mechanical Engineering, Manufacturing Technology Laboratory, National Technical University of Athens, Heroon Polytehniou 9, Athens, 15773 Greece; 3 Faculty of Electrical and Control Engineering, Gdańsk University of Technology, 11/12 Gabriela Narutowicza Street, 80‑233, Gdańsk, Poland

**Keywords:** Human activity recognition, Manufacturing, Gestures, Motion recognition, Sensors, Artificial neural networks, Deep learning, Classification, Convolutional neural network, Feedforward neural network, Mechanical engineering, Computer science

## Abstract

This article addresses the challenge of human activity recognition (HAR) in industrial environments, focusing on the effectiveness of various neural network architectures. In particular, simpler Feedforward Neural Networks (FNN) are focused on with an aim to optimize computational performance without compromising accuracy. Three FNN configurations—FNN1, FNN2, and FNN3—were evaluated alongside the Convolutional Neural Network (CNN 1D) model for comparative analysis. The results indicate that the FNN achieved accuracy rates ranging from 94.28 to 99.19%, while the CNN 1D exhibited an accuracy of 98.12%. Despite the CNN 1D’s efficiency for real-time applications, the FNN’s fast training times and high accuracy make them particularly valuable in resource-constrained environments such as mobile devices. The findings suggest that while more complex models such Long Short-Term Memory (LSTM)-Auto-Encoder configurations, that have been tried by the same research group before, may offer better adaptability, simpler architectures can provide effective results in HAR tasks. Notably, these simpler models can be adopted in cascading systems operating online, serving as detectors of known activities for real-time monitoring and classification.

## Introduction

The challenge of Human Activity Recognition (HAR) encompasses the automatic identification and classification of human activities using various types of sensory data. The primary objective of HAR is to map sequences of input data to specific labels that represent distinct human activities. Addressing the HAR problem is crucial across numerous fields, contributing to the advancement of sophisticated monitoring systems, behaviour analysis, and human-technology interaction. Solutions of this problem are applicable across multiple domains, including monitoring physical activities such as running, cycling, and swimming; tracking patient activities; analysing actions associated with operating industrial machinery; and detecting specific postures and movements involved in driving a vehicle.

HAR algorithms must tackle challenges such as processing time-series data, learning features that distinguish individual activities, managing limited training data, and adapting to diverse scenarios and usage conditions. The data involved in HAR can be highly complex due to the diversity of activities, environmental conditions, individual variability, and noise and interference pertaining to the sensors employed.

This paper focuses on the recognition of worker activities at the production level, typically involved in assembly and disassembly, that are characterized by considerable complexity. This complexity stems from the large number of distinct tasks that workers perform, the varying durations of these tasks, and the inconsistent rate at which they are executed.

In HAR problems typical input data include signals from sensors worn by individuals^1–4^ such as smartphones, smartwatches, fitness bands, or sensors embedded in clothing. These data usually encompass information on acceleration, speed, rotation angle, and other physical parameters. Some HAR studies incorporate multimodal data, such as those from cameras and microphones, alongside inertial sensor data and radio frequencies, which can enhance the effectiveness of movement classification^5,6^.

In this study, wearable sensors were selected for generating input data due to their ease and speed of signal transmission, lower computational cost compared to analysing video or images, and the wireless connection of the signal recorder to the robotic mechanism, among other factors.

Various approaches, techniques, and challenges related to human activity recognition are discussed in the works^7^. The surveys give the summary of different types of machine learning techniques, covering Decision Trees, K-Nearest Neighbours, and Support Vector Machine techniques, Hidden Markov models and Neural Network Techniques such as Artificial Neural Network (ANN), Convolutional Neural Network (CNN) and Recurrent Neural Network (RNN).

The analysis of existing achievements presented in the literature highlights a proportional relationship between time complexity and accuracy. However, this article presents models with relatively low complexity that can achieve comparable results to those of very high-complexity models. Additionally, the size of the data, the type of sensors, and their placement also affect the results. The paper^8^ reviews recent advancements in deep learning for sensor-based activity recognition, summarizing the existing literature from three key perspectives: sensor modalities, deep learning models, and their applications. Further literature reviews^1,9^ indicate that neural networks, particularly CNN and RNN, are promising tools for addressing the HAR problem, achieving high accuracy in classifying human movements when the network architecture is appropriately tailored to the specifics of the input data and task requirements. RNNs are utilized in HAR tasks due to their ability to model temporal dependencies in data sequences; however, they are characterized by high complexity. RNN-based models, such as LSTM and Gated Recurrent Unit (GRU), are commonly employed for processing movement sequences and consistently yield competitive performance in human activity classification^10,11^.

Some studies utilize hybrid models that combine features of different types of networks, such as integrating CNNs with RNNs, to enhance input data representation, improve learning efficiency, and facilitate sensor data fusion. Authors of the article^6^ used a time-series multimodal architecture that combines GRU cells with CNNs to handle accelerometer and gyroscope data. In^12^, the authors proposed a hybrid deep neural network that integrates a hierarchical CNN and RNN to analyze daily activities of higher complexity. This approach utilizes multiple Inertial Measurement Units (IMUs) placed on specific parts of the body, each acting as a distinct sensor with varying sampling frequencies. For input sequence data consisting of short time series^5^, introduced a deep neural network architecture combining convolutional, recurrent, and softmax layers. A Feedforward Neural Network processes information through a series of interconnected computational nodes. The nodes in the layers transform the data using nonlinear operations, creating a decision boundary by projecting the data into a space where it becomes linearly separable^13^.

Multi-layer perceptrons (MLPs) have been successfully applied to a variety of tasks, including data prediction^14^, modelling^15^, and classification^16^, through supervised training. The study in^17^ introduces a novel approach for interpreting and characterizing the performance of the FNN rate of penetration prediction (ROP) model using the Rectified Linear Unit (ReLU) activation function. The curve representing the relative activation frequency of neurons in the hidden layer provides valuable insights into overfitting in the FNN ROP model and facilitates the assessment of drilling data similarity. The results demonstrate that this FNN prediction model, utilizing the ReLU activation function, performs exceptionally well. Several papers uses FNNs in HAR problems because of their capability to map complex inputs to outputs. However, this is an unusual approach for this type of problems and requires ingenious modelling the problem to fit into the underlying information representation supported by FNNs^18^. analyzes the performance of FNN architectures in HAR applications. This paper shows that the choice of classifier is crucial for the effectiveness of HAR with FNNs. In a supervised learning process, actions are initially represented using hand-crafted features, and then a classifier is trained to perform the classification. It was observed that FNNs can be effectively applied to HAR problems, even in tasks where sequential dependencies between input data and outcomes are crucial^19^^20^. compared performances of FNN and cascade forward neural network for face recognition with principal component analysis to extract the features. The effectiveness of CNN and FNN methods was compared in recognizing hand gestures^21^. The hand gesture recognition framework using FNN yielded better results than CNN in terms of performance metrics, with less data loss and higher accuracy. The authors report prediction accuracies of 84.1% for FNN and 77.8% for CNN, respectively.

The HAR problem addressed in this paper was initially studied in^11^. To automate the recognition of tasks performed by workers engaged in typical activities related to manual parts assembly and handling within manufacturing cells, a deep learning-based approach was employed. Two types of neural networks were used to classify these activities: a variational autoencoder neural network and an LSTM network, achieving an accuracy of approximately 80%. Despite this relatively high accuracy and the potential for online application, this approach requires a computationally intensive system, leading to prolonged training and data processing times when using the same volume of training data.

The literature review indicates that no single method is optimal for recognizing human activities. To select the most suitable method for a specific application, various factors must be considered, and the approach should be adjusted accordingly. Drawing from insights gained in previous studies, the primary focus of this work was to evaluate the effectiveness of FNN architectures in the context of the HAR problem. Specifically, we chose to utilize the same dataset as in the prior study in article^11^, but we explored networks of lower complexity, examining three different configurations of FNNs designated as FNN1, FNN2, and FNN3. The parameters defining the architectures of FNN1 to FNN3 significantly influence their performance and accuracy. In addition to FNNs, a CNN 1D model was implemented for comparison purposes, leveraging its known ability to effectively process sequential data, such as time series or signals (see comprehensive surveys in the papers^14,22^).

Online classification in neural networks refers to the model’s capability to process data in real time, adapting to new samples without the need to retrain the entire network. In the context of online classification using FNN or CNN, the weights can either remain fixed or be updated continuously.

In the first approach, the neural network is trained once on a previously collected dataset, and upon completion of the training phase, the weights are retained as fixed during online classification. The model operates solely as a classifier, accepting successive samples or time window segments and making predictions based on the established weights. This approach is employed when it is assumed that the underlying relationships described by the data will not significantly change over time, provided the model adequately represents the data and no changes in the data distribution are anticipated.

In cases of online classification with adaptation, weights can be updated continuously through methods such as mini-batch learning or incremental learning. This dynamic learning approach is advantageous when user activity changes or when new data patterns emerge. While this solution offers greater flexibility, it requires additional computational power. Offline models necessitate less computational power post-deployment, which may be significant in resource-constrained devices such as smartphones or wearable technology (e.g., smartwatches).

In this study, we analysed four types of networks (FNN1, FNN2, FNN3, and CNN 1D) that can function as classifiers and be applied in an online mode alongside an offline model. The network models were trained on a previously collected and comprehensive dataset representing a range of typical activities, such as: using a screwdriver, using a wrench, part picking, part placing and surface cleaning. Upon completion of the training phase, the network weights were fixed and remained unaltered during prediction, eliminating the need for additional learning operations. The FNN1 and FNN2 networks recognized activities, while the FNN3 and CNN 1D networks identified specific phases of these activities.

Thus, the main objective of this work is to employ much simpler neural networks than those commonly used to tackle HAR problems (FNN, CNN 1D), while leveraging appropriate problem modelling techniques to achieve high recognition performance. Indeed, the novelty of the approach was demonstrated by the improved accuracy achieved in activity recognition across all tested models, with results ranging from 97 to 99%. The challenges related to the palette of human worker activities to be recognized, data collection methods, and the raw data collected are presented in Sect. 2. The data preparation process is detailed in Sect. 3. The FNN and CNN 1D models are described in Sect. 4, along with their results. A discussion of the recognition performance achieved and the potential for addressing general subtypes of HAR problems is provided in Sect. 5. Conclusions and future directions for research are articulated in Sect. 6.

## Human worker activities and their recording

The original dataset used in this work stems from previously reported work by the same authors^11^, which the reader is referred to for details.

### Activities and phases

**Fig. 1:**
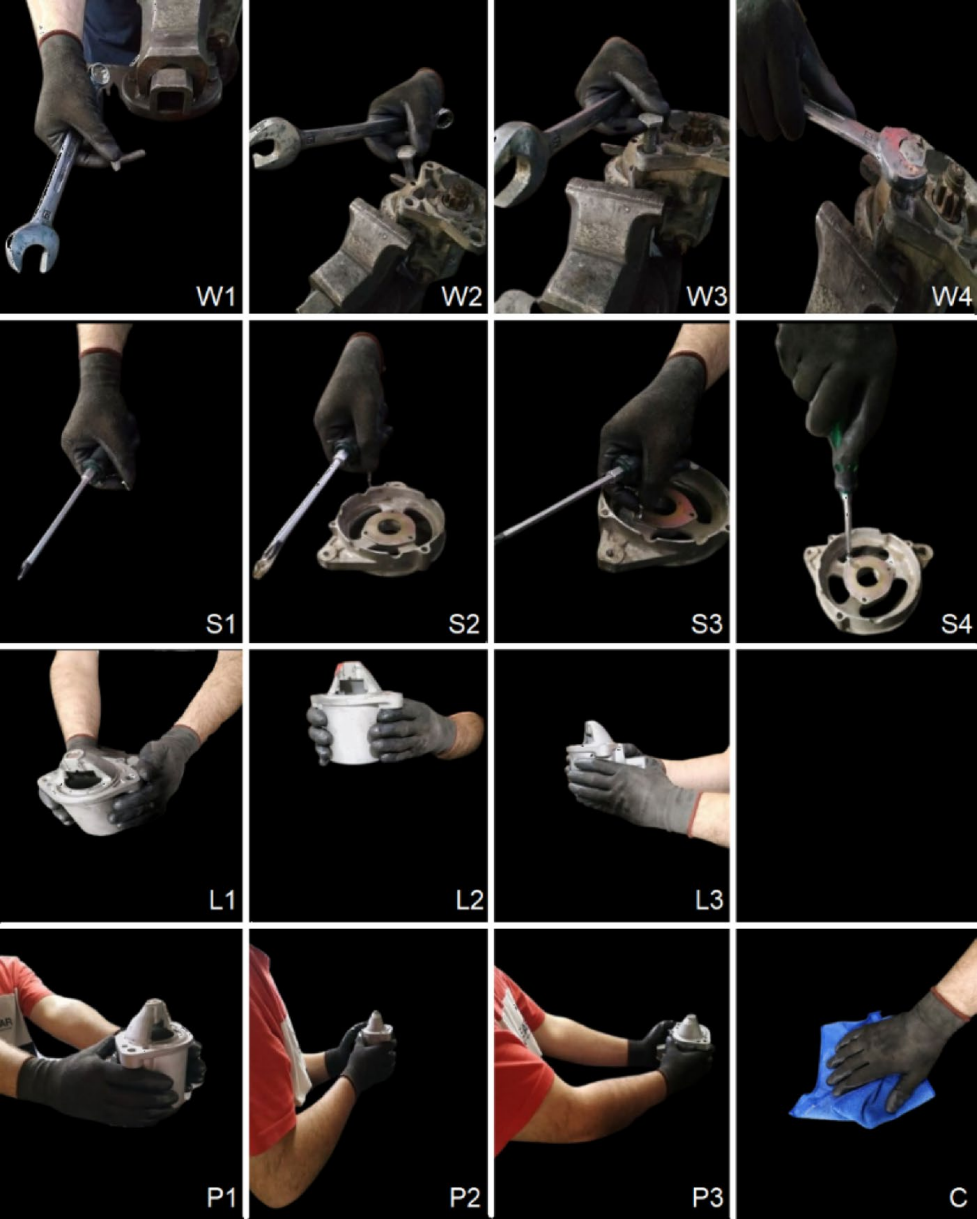
Activity depiction in terms of phases, where applicable.

Five common industrial worker activities were selected as representative ones to demonstrate the methods employed, as summarized in Table 1. Characteristic snapshots are shown in Fig. 1.

**Table 1:** Human worker activities tackled.

No.	Activity	Acr	Phases	Objects	Motion patterns	Dur’*n* (sec)
1	Screwing	S	S1 Grasping & lifting the screw S2 Moving screw above hole S3 Putting screw in hole S4 Screwing with screwdriver	Screw Screwdriver Hole	Coarse picking movements Subtle placing movements Repetitive wrist rotations	8
2	Wrenching	W	W1 Grasping & lifting the bolt W2 Moving bolt above hole W3 Putting bolt in hole W4 Bolting with wrench	Bolt Wrench Hole	Coarse picking movements Subtle placing movements Repetitive arm rotations	8
3	Part Lifting	L	L1 Part grasping by extended hands L2 Part lifting near body L3 Body directing to destination	Part Bench	Fast grasping movements Slow lifting movements Hands still during transfer	5.5
4	Part Picking and Placing	P	P1 Part grasping by extended hands P2 Part holding near body P3 Part placing onto destination	Part Surface1 Surface2	Fast picking movements Slow placing movements Hands still during transfer	5.5
5	Surface Cleaning	C	Cleaning motion	Cloth Surface	Trochoidal or reciprocal hand motion parallel or normal to body	5.5

More specifically, each activity except Surface Cleaning (S) consists of 4 or 3 constituent phases. Some activities involve, as a main phase, characteristic motions, see Table 1, e.g. repetitive motions such repetitive wrist rotation for activity Screwing (S) involving a screwdriver, repetitive arm rotations for activity Wrenching (W) involving a key or wrench and reciprocal or trochoidal hand movement for activity Surface Cleaning (C). In other activities, notably Part Lifting (L) and Part Picking and Placing (P) there are no such main phases that can uniquely identify the whole activity to which the phase belongs. In addition, there are also secondary phases such as picking the screw or the bolt and the respective tool before using it, which have different motion characteristics than the main phase. These can be considered to reinforce identification of an activity when considered in addition to the characteristic main phase. Yet, in the absence of a main characteristic phase, a sequence of secondary phases may well provide unique identification of an activity. In any case, motion kinematics are key to recognizing movement related activities, as is also reasonably expected.

Note that there are many possible variations in performing these activities. These refer not only to the speed of execution but also to spatial parameters such as the height of the surface from which a part or a tool is grasped and similarly of the surface onto which it is placed. To cover such variability, three different surface heights were tried in activities of type L and P. Motion patterns may also differ in activities such as cleaning (type C). Three different patterns were executed in this case, too, i.e. movement of the hand holding the cleaning cloth parallel to the worker’s body, normal to it as well as a trochoidal pattern which is quite commonly employed, too. The size and weight of the parts and the tools is also expected to affect the kinematics of the pertinent activity, but these were kept the same throughout the experiments.

### Sensors and signals

Execution of an activity is monitored in terms of a bundle of signals according to Arduino Science Journal application^23^ running on a smartphone. This application was used to record 6 kinematics related signals pertaining to acceleration and orientation and another two extra signals. Note that gyroscope signals were not recorded, thus angular velocity was not captured. These signals are directly related to the motions performed by the worker, holding information that is directly required for activity recognition. These are:

- x-axis acceleration (AccX - m/s^2^).
- y-axis acceleration (AccY - m/s^2^).
- z-axis acceleration (AccZ - m/s^2^).
- total linear acceleration excluding gravity (Linear Accelerometer Sensor [LAS] - m/s^2^)
- compass (Compass Sensor [CS] - deg with respect to earth’s north pole)
- geomagnetic field strength (Magnetic Rotation Sensor [MRS] - µT).

The latter is often used in an e-compass mode complementing or replacing the compass, which is often GPS-based. Note that acceleration z axis is out of the phone’s screen, normal to it, y is from bottom to top along the screen’s height and x is from left to right.

Non kinematic signals include:

- brightness (exposure) of the front camera (BEV).
- sound level (Decibel Source Sound [DSS] db).

In general, it is not clear beforehand which sensors hold most valuable information for each activity hence all of them are given equal focus. However, there may be additional characteristics that can provide valuable clues, such as:

- sound, e.g. in case of surface cleaning by rubbing a cloth against a surface.
- crude vision characteristics, e.g. brightness of the image of the camera indicating, e.g., whether the human hand is oriented towards the workbench (darker) or towards the ceiling (brighter),
- strength of the magnetic field, which in addition to indicating orientation with respect to north pole, is also heavily affected by presence of metal objects or other magnetic fields, such as motors, thus indicating closeness of metal hand tools or even large metal surfaces.

The smartphone was tied to the wrist of the worker because this location was not intrusive in any of the activities studied and also signal changes, especially acceleration, were more pronounced at the wrist in comparison to other parts of the workers’ body. In fact, two workers were monitored performing in total 125 runs of each of the 5 activities.

**Fig. 2:**
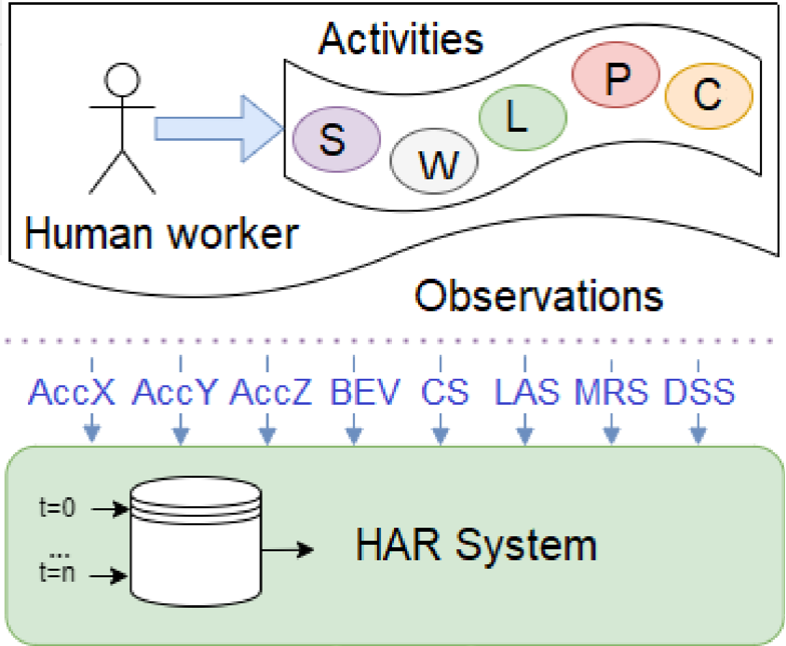
Generalized schema of human activity observations for HAR system.

Sensor values are recorded at a sampling frequency of 15 Hz. These values were collected in a database with time-stamping and were used to train the HAR system, see Fig. 2.

**Fig. 3:**
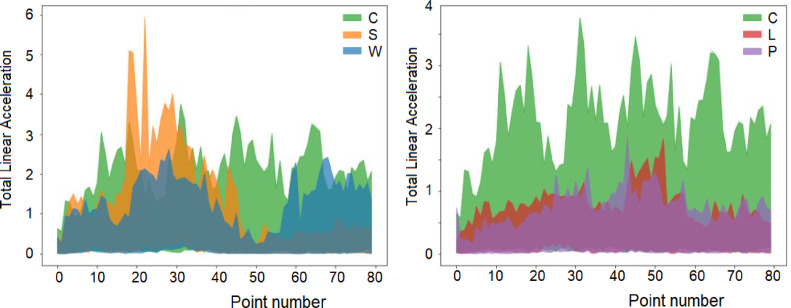
Total acceleration observation values for all activities.

The range of values of total acceleration for all 625 signals collected (125 per activity) is presented in Fig. 3, where significant overlap is immediately obvious, which renders the direct distinction of individual activities impossible by visual inspection.

The sensor data has been appropriately prepared, as described in Sect. 3 next, to allow training of basic yet effective neural networks of low computational load, as outlined in Sect. 4.

## The dataset

The HAR problem described can be distilled into a classification problem involving categorizing data into predefined classes (activities) as well as further segmenting these activities into distinct phases or stages. Four approaches were followed and compared to assess their effectiveness in classification and segmentation. All networks were fed the same signals as inputs for training and testing, which form the complete dataset. This is described next.

Each one of the 5 activities was executed 125 times making for 625 executions or observations. Each execution is recorded by 8 sensors namely: AccX, AccY, AccZ, BEV, CS, LAS, MRS, DSS, in 80 sequential time points, *j = 1.80*. All signals from the 8 sensors are simultaneously input into the network, forming a total 640 input neurons.

The number of points into which the signals of an activity are discretised is determined by the product of the activity duration and the sampling rate. The screwing and wrenching activities (S, W) lasted approximately 8 s whilst the rest (C, L,P) only about 5.5 s. However, this difference was eliminated by truncating activities S and W due to the underlying repetitive motion pattern. Thus 80 points (5.5 s X 15 Hz) were uniformly adopted for discretization of all activity signals.

The set of all observations is denoted $\:{\varvec{X}=\left[{x}_{1}||,{x}_{2},\dots\:{x}_{i}||,\dots\:,{x}_{N}\right]}^{T}$. Observation $\:{x}_{i}$. where *i=1.N*, *N=625* corresponds to one activity, namely: S, W, L, P, C.

$$\:{x}_{i}=\left[{AccX}_{1},{AccX}_{2},{AccX}_{j}...,{AccX}_{80},...,{DSS}_{1},{DSS}_{2},{DSS}_{j}...,{DSS}_{80}\right]$$

**Fig. 4:**
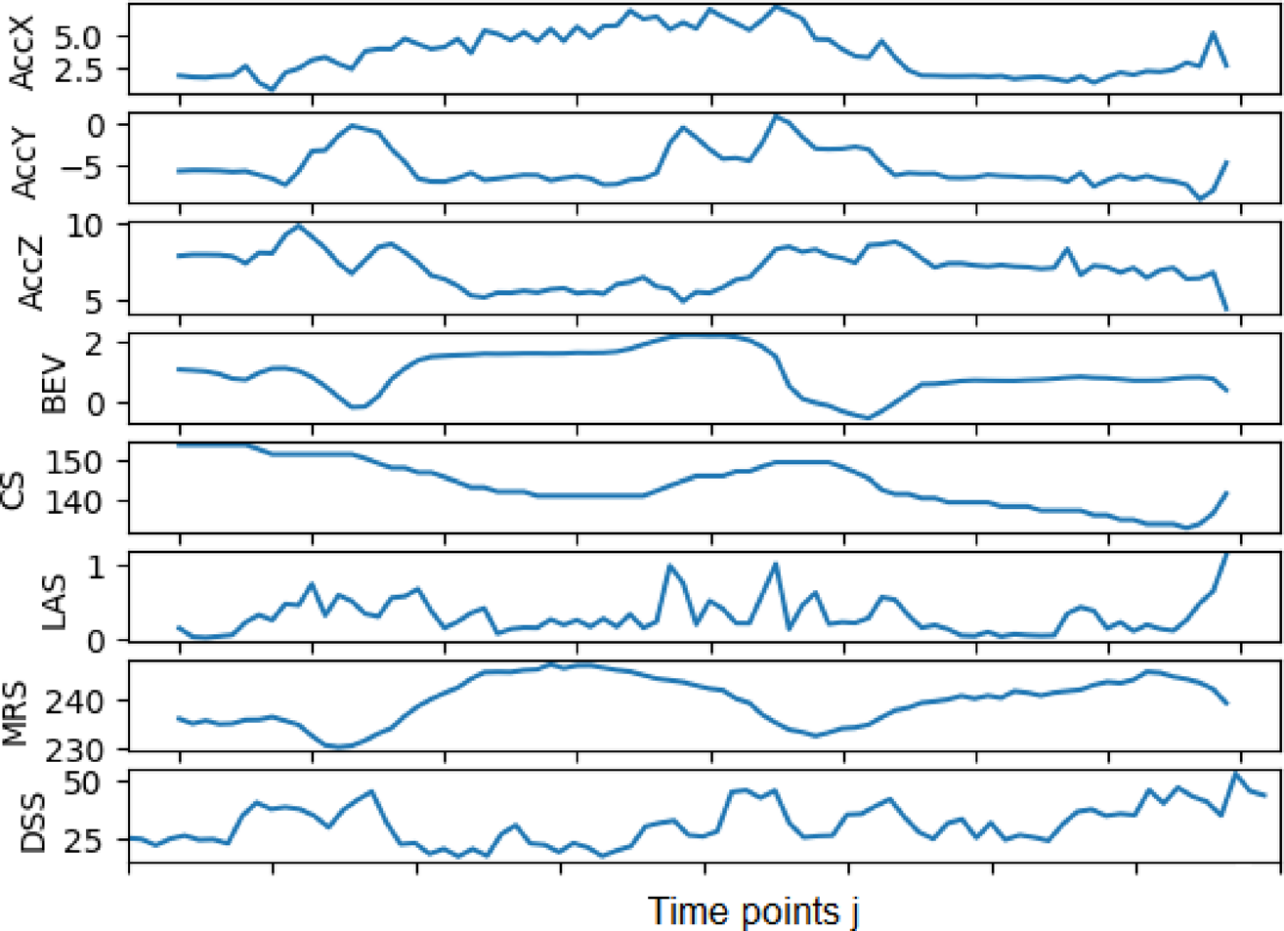
Typical input values for W activity execution-observation $\:{\text{x}}_{\text{i}}$ to the network.

Figure 4 shows an example of one sample input to the network for activity W (Bolting with a wrench).

The values fall into time intervals of varying ranges. To eliminate the differences, a standard score scaling^24^ process was applied to the input values and the expected output values. The mean $\:\mu\:$ and standard deviation $\:\sigma\:$ of all observed values are known, so the raw score $\:\varvec{X}$ can be converted to a standard score using the formula

$$\:{x}_{i}^{*}=\frac{{x}_{i}-\mu\:}{\sigma\:}$$

The training set, consisting of 500 observations, was utilized for training the model. The test set included 125 observations and was created to assess the model’s performance. This meticulous preparation of data sets ensures a systematic and well-organized approach to subsequent stages of model training and evaluation.

## The neural networks constructed

This chapter presents four alternatives to activity classification, differing in both structure and the method of generating outputs. First, three FNNs are presented differing in various aspects as presented next. The main metrics to evaluate training and testing performance are: Mean Squared Error (*MSE*), $$\:MSE\left(y,{y}_{pred}\right)=$$ $$\frac{1}{n}{\sum\:}_{i=0}^{n-1}{\left({y}_{i}-{y}_{{pred}_{i}}\right)}^{2}$$ and *Mean Absolute Error (MAE),* $$MAE\left(y,{y}_{pred}\right)=\frac{1}{n}{\sum\:}_{i=0}^{n-1}\left|{y}_{i}-{y}_{{pred}_{i}}\right|$$, both utilising the difference between the expected values $$\:y$$ and the output values $$\:{y}_{pred}$$. This was supplemented by $$\:Precision\left(y,{y}_{pred}\right)=TP/(FP+TP)$$, $$\:Recall\:\left(y,{y}_{pred}\right)=TP/(FN+TP)$$, and $$\:F1-score\:\left(y,{y}_{pred}\right)=\frac{2*precision*recall}{precision+recall}$$, $$\:Accuracy\left(y,{y}_{pred}\right)=(TN+TP)/(TN+FP+TP+FN)$$ which are typically used to assess the performance of classifier models^25^, where *TP/TN* denote the number of true positive/negative predictions and FP/FN denote the number of false positive/negative predictions.

Then a CNN 1D is also constructed, as this is typically used to tackle HAR problems and is employed for comparison of the FNNs. The architecture of the four networks is summarised in Table 2. The network parameters were determined empirically, based on a trial-and-error approach. A key factor considered was the simulation time—network parameters were adjusted to enable the best possible comparison of achieved results. The starting point was the establishment of parameters for FNN1 because FNN1 is harder to train than the rest FNNs as it has the lowest number of trainable parameters. Note that in this trial-and-error exercise we did not maintain detailed documentation of all analysed configurations, because the primary objective of our study was to demonstrate the feasibility of using FNNs in HAR rather than to optimize FNN architecture.

For a smaller number of neurons and number of hidden layers, worse results were obtained. Particulars as well as training specifics and results achieved are presented in the next sub-sections. Note that the input layer in all cases consists of 80 time points x 8 sensor values per point = 640 sensor values.

**Table 2:** Summary details on the architecture of the alternative neural networks employed.

NN type	FNN1			FNN2			FNN3			CNN1D		
Layer Number	Layer Type	Output Shape	Param	Layer Type	Output Shape	Param	Layer Type	Output Shape	Param	Layer Type	Output Shape	Param
1	Dense	50	32,050	Dense	50	32,050	Dense	50	32 050	Conv 1D	13,8	520
2	Dense	50	2,550	Dense	50	2,550	Dense	50	2 550	MaxPool 1D	6,8	0
3	Dense	50	2,550	Dense	50	2,550	Dense	50	2 550	Flatten	48	0
4	Dense	50	2,550	Dense	50	2,550	Dense	50	2 550	Dense	50	2550
5	Dense	100	5,100	Dense	100	5,100	Dense	100	5 100	Dense	50	2550
Output	Dense	1	101	Dense	5	505	Dense	15	1515	Dense	15	765
Total Params	44,901 (175.39 KB)			45,305 (176.97 KB)			46,315 (87.17 KB)			6385 (24.55 KB)		
Trainable Params	44,901 (175.39 KB)			45,305 (176.97 KB)			46,315 (87.17 KB)			6385 (24.55 KB)		

### Activity recognition with FNN1 and FNN2

**Fig. 5:**
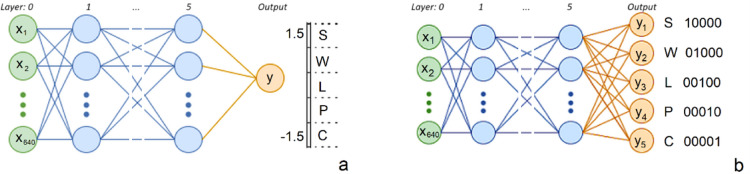
Architecture of (**a**) FNN1 (**b**) FNN2.

The first approach involves constructing a simple regression FNN, where a single real-valued output is generated and compared to the actual class label, as shown in Fig. 5(a). After the data normalization process, the output signals from the network were transformed and now range from − 1.5 to 1.5. The model utilizes the ReLU activation function in the hidden layers and a linear activation function in the output layer.

**Fig. 6:**
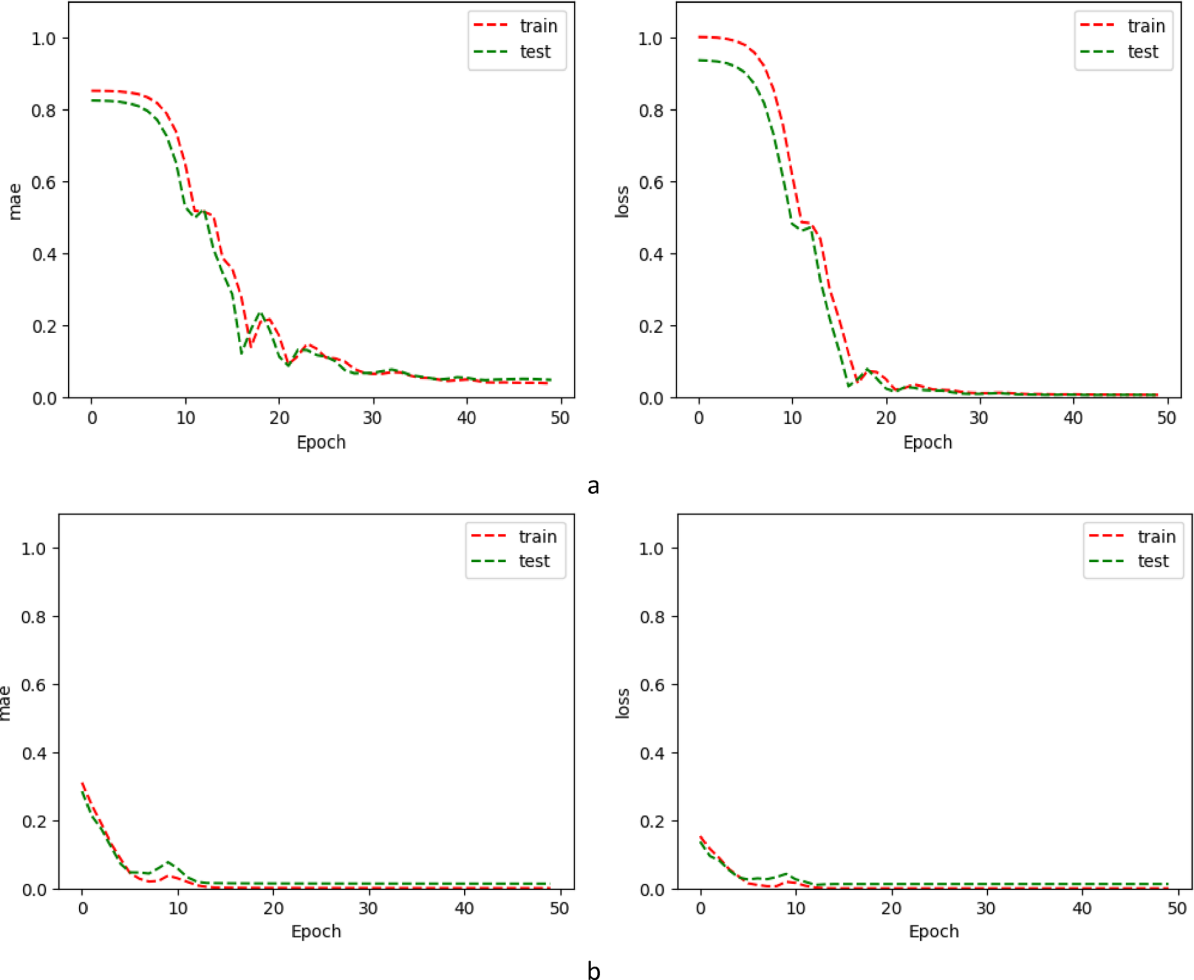
Loss and Metric Value evolution through the training and testing process for (**a**) FNN1 (**b**) FNN2.

The Adam optimizer^26^ was used in the training process, along with the MSE loss function. The training progress (with metric function MAE), depicted in Fig. 6(a), illustrates the evolution of the loss and the metric function. The both function values approaching zero early in the training process and remaining stable throughout subsequent epochs indicate the model’s high effectiveness in predicting the expected results.

**Fig. 7:**
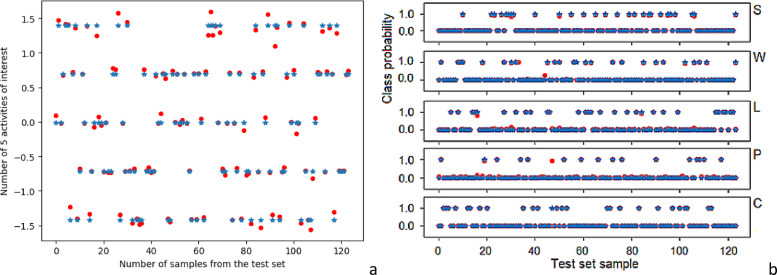
Graph of output (red) and expected (blue) activities in the testing process for (**a**) FFN1 (**b**) FFN2.

The results on the test dataset are shown in Fig. 7(a). The blue values represent the expected outputs, while the red dots indicate the values produced by the network. The conducted network tests demonstrate that the trained model is highly suited for the intended objectives. The best results were achieved for class Part Lifting (L), which is clearly visible on the chart – the output values closely align with the expected values, showing excellent model performance.

A significant advantage of the first approach, FNN1, is its low complexity, which contributes to a rapid training time for the network. The conducted tests demonstrated that the output values are very close to the expected values, facilitating easy class identification. In the case of the second approach, FNN2, equally impressive classification results were observed. Furthermore, this approach provides a much clearer separation of classes. The same type of FNN was employed, with the key difference from FNN1 being the number of outputs as shown in Fig. 5(b), as detailed in Table 2. This is a classic method used in classification problems, based on the assumption that the most active output identifies the class.

In both cases, the Adam algorithm was utilized for training the network, analogous to backpropagation, along with the MSE as the loss function and MAE as the metric functions, as illustrated in Fig. 6(b). The output values are interpreted as probabilities that the activity under consideration belongs to specific classes, as shown in Fig. 7(b). The model achieves correct predictions for approximately 99% of instances in the evaluation set, indicating excellent performance and confirming the model’s adequacy. Note that, in both FNN1 and FNN2, activity phases have not been taken into consideration, this possibility being exploited in FNN3 and CNN-1D.

### Phase recognition with FNN3

**Fig. 8:**
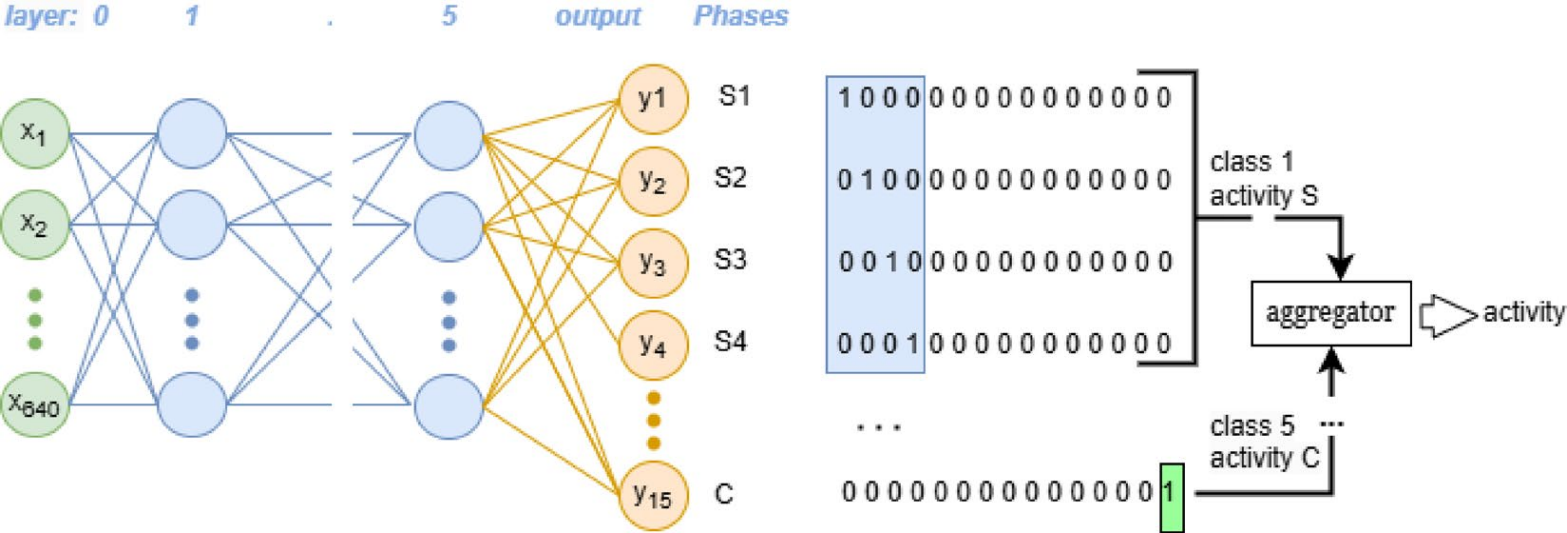
Architecture of FNN3.

The third approach considers the constituent phases of each activity in order to increase identification accuracy. Individual activities were divided into phases, which were to take place in a specific order. FNN has been employed for binary classification with fifteen outputs, which is the sum of the phases identified in the five activities examined, see Table 1. The relevant architecture is depicted in Fig. 8.

**Fig. 9:**
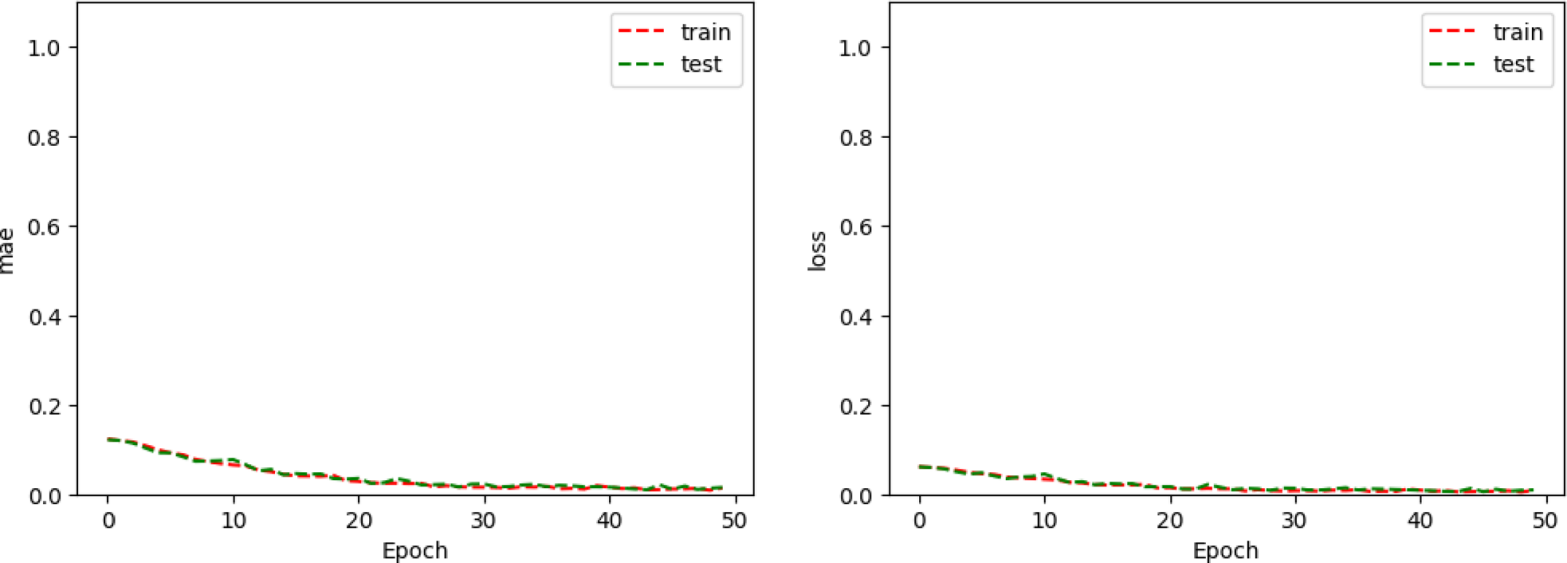
Loss and Metric Value evolution for FNN3 throughout the training and testing process.

In this approach, the training process comprised 100 iterations, with the initial weights set randomly. The graphical representation (see Fig. 9) illustrates a clear reduction in error throughout the training and testing process. A key aspect of this methodology is the individual recognition of each phase during network training.

It was only at the operational stage of the network that an aggregator was applied, which facilitated the classification of a complete activity composed of sequential phases. The network output exhibiting the highest level of activity during operation indicated that the corresponding phase had been successfully recognized. The classification of the complete activity became feasible only after the implementation of the aggregator, which confirmed whether the recognized phases were arranged in the correct order.

The network was subsequently tested, yielding very favourable results. The model makes correct predictions for approximately 94,28% of the instances in evaluation set. This approach proved to be the best in terms of accuracy but also the most complex in terms of the number of parameters.

### Phase recognition with CNN-1D

**Fig. 10:**
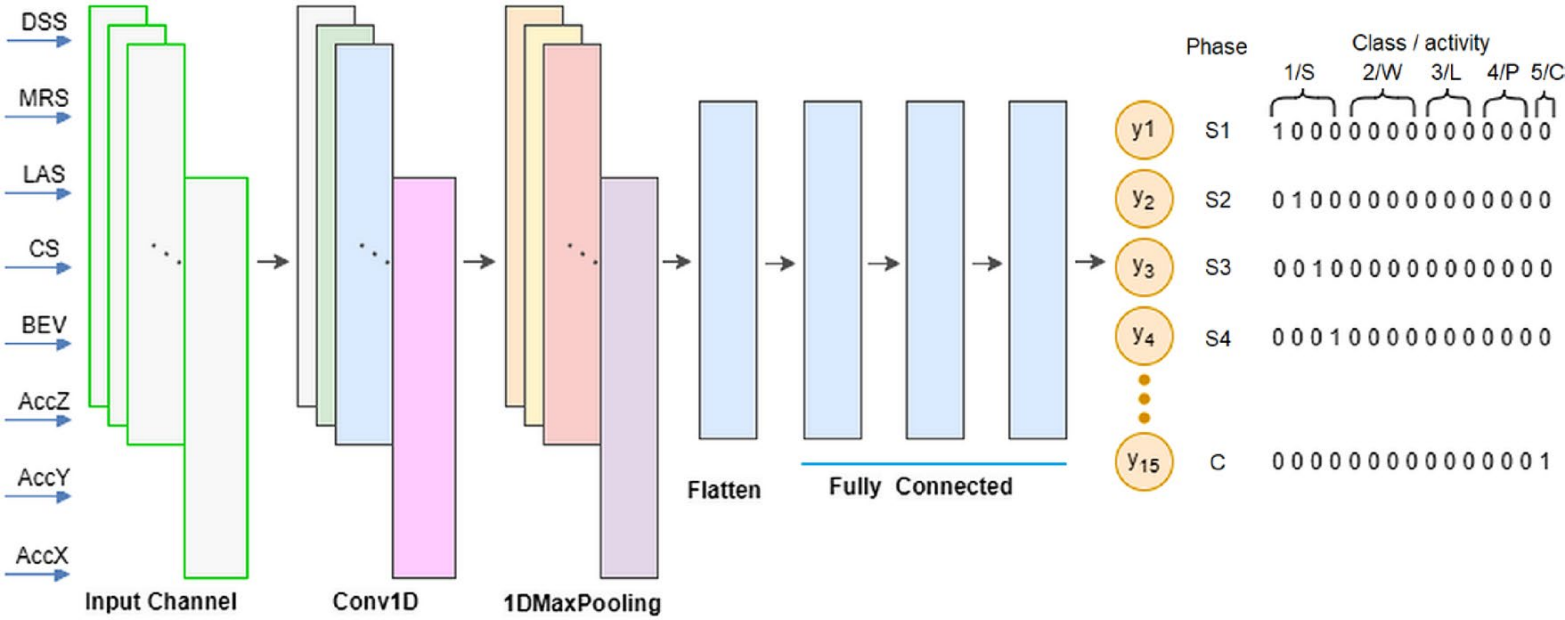
Architecture of the CNN1D.

This model comprises a combination of convolutional (Conv 1D) and dense layers for binary classification of phases with 15 outputs as shown in Fig. 10. The same data were fed into the network inputs, in accordance with previous approaches. The model architecture and key details are summarized in Table 2 and in Fig. 10.

**Fig. 11:**
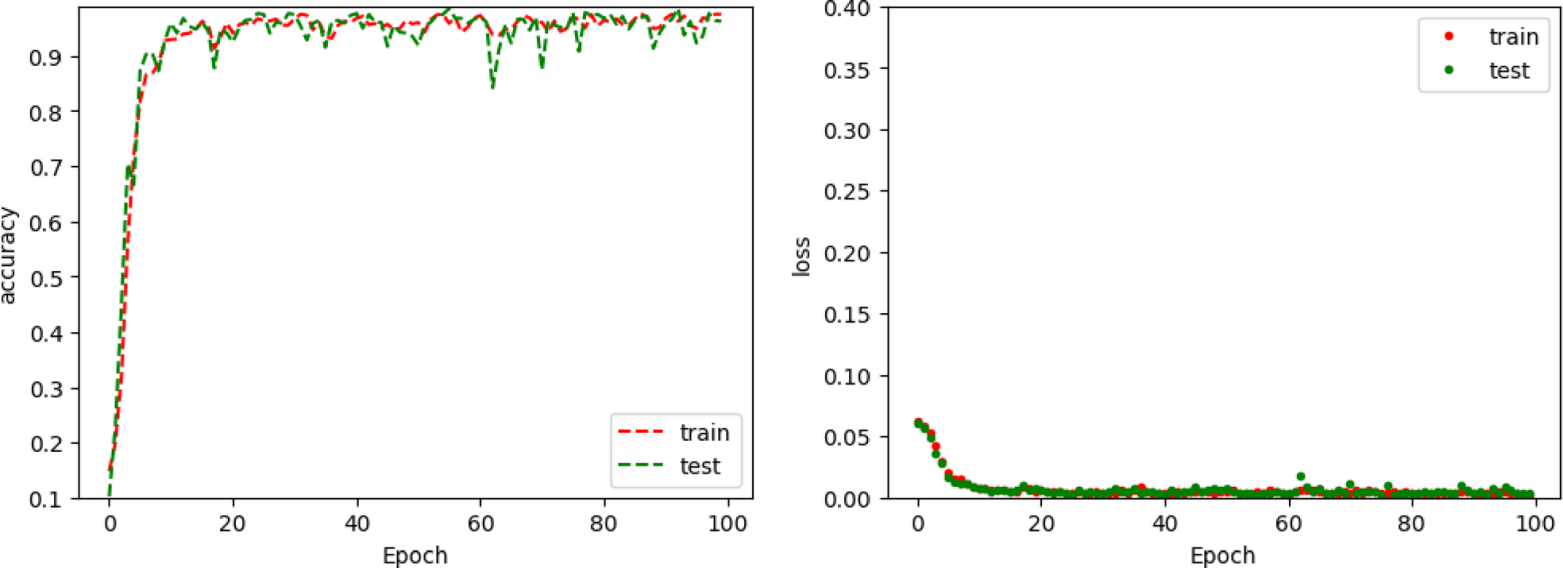
Loss and Metric Value evolution for CNN-1D throughout the training and testing process.

The loss value attained after training was minimal, i.e. 0.003 and the reported accuracy value in the testing process was 0.971 (see Fig. 11).

Table 3 displays high values along the diagonal, indicating that the CNN-1D effectively recognizes individual activity phases, with minimal classification errors (e.g., S4 as S3 or W3 as W4). These minor errors suggest that while the CNN-1D performs well in classification, some phases may exhibit similarities. Given the absence of class imbalance, accuracy serves as an appropriate evaluation metric. Overall, the CNN-1D demonstrates high effectiveness for this dataset, and its ability to capture temporal dependencies in sequences may further reduce minor classification errors and improve overall activity classification accuracy.

**Table 3:** Confusion matrix for activity classification using CNN-1D model.

Phase	S1	S2	S3	S4	W1	W2	W3	W4	L1	L2	L3	P1	P2	P3	C
S1	**29**	0	0	0	0	0	0	0	0	0	0	0	0	0	0
S2	0	**18**	0	0	0	0	0	0	0	0	0	0	0	0	0
S3	0	0	**25**	0	0	0	0	0	0	0	0	0	0	0	0
S4	0	0	**2**	**26**	0	0	0	0	0	0	0	0	0	0	0
W1	0	0	0	0	**30**	0	0	0	0	0	0	0	0	0	0
W2	0	0	0	0	0	**23**	0	0	0	0	0	0	0	0	0
W3	0	0	0	0	0	0	**24**	**1**	0	0	0	0	0	0	0
W4	0	0	0	0	0	0	**3**	**23**	0	0	0	0	0	0	0
L1	0	0	**1**	0	0	0	0	0	**25**	0	0	0	0	0	0
L2	0	0	0	0	0	0	0	0	0	**29**	0	0	0	0	0
L3	0	0	0	0	0	0	0	0	0	0	**15**	0	**1**	**3**	0
P1	0	0	0	0	0	0	0	0	0	0	0	**26**	0	0	0
P2	0	0	0	0	0	0	0	0	0	0	0	0	**27**	0	0
P3	0	0	**1**	0	0	0	**1**	0	0	0	0	0	0	**21**	0
C	0	0	0	0	0	0	0	0	0	0	0	0	0	0	**18**

The analysis of classification (see Table 4) indicates that the CNN 1D model achieves high accuracy across all five activities, with slight variations. Activity S has an accuracy of 98%, reflecting good overall model performance despite minor misclassifications, likely arising from overlapping features with other activities. Activity W achieves an accuracy of approximately 96.15% and L-97,18%, P-97,37% with several misclassifications also suggesting shared characteristics with other activities. In contrast Activity C demonstrate perfect accuracy (100%), indicating that this activity possess distinct features that the model reliably recognizes. This suggests that while the model is highly effective in distinguishing clear patterns, it could benefit from further refinement to better handle activities with overlapping characteristics, particularly in the case of Activities S and W. Overall, the model exhibits solid performance and is well-suited for accurate activity classification tasks, although further improvements in feature extraction could enhance its accuracy for closely related activities.

**Table 4:** Summary of classification metrics for each activity using the CNN-1D model.

Activity	TP	FP	Acc
S	98	2	98%
W	100	4	96.15%
L	69	0	97,18%
P	74	0	97,37%
C	18	0	100%

## Discussion

The aim of constructing alternative neural networks was to evaluate the effectiveness of different neural network architectures in the context of activity classification and to identify which approach best meets the requirements for accuracy and computational load. They were also constructed to enable comparison of the results with those presented in other studies following a much more elaborate approach using AutoEncoder and LSTM networks^11^.

Performance of the different models constructed is summarised in Table 5.

**Table 5:** Summary details on the performance of the alternative neural networks employed.

NN type	FNN1	FNN2	FNN3	CNN1D
Training time (ms)	5577	5806	6348	8469
Execution time (ms)	4	5	5	3
Training performance (MSE)	0.005	0.012	0.009	0.003
MAE	0.047	0,014	0.015	-
Accuracy	99.19%	99.19%	94.28%	98.12%
Precision	S: 100% W: 96.43% L: 100% P: 100% C: 100%	S: 100% W: 96% L: 100% P: 100% C: 100%	S: 92.17% W: 100% L: 91.89% P: 98.44% C: 76.19%	S: 98.04% W: 99.05% L: 100% P: 94.87% C: 100%
Recall	S: 100% W: 100% L: 95.83% P: 100% C: 100%	S: 100% W: 100% L: 95.65% P: 100% C: 100%	S: 100% W: 95.88% L: 90.67% P: 87.50% C: 94.12%	S: 100% W: 100% L: 93.24% P: 97.37% C: 100%
F1-score	S: 100% W: 98.18% L: 97.87% P: 100% C: 100%	S: 100% W: 97.96% L: 97.78% P: 100% C: 100%	S: 95.93% W: 97.89% L: 91.28% P: 92.65% C: 84.21%	S: 99.01% W: 99.52% L: 96.50% P: 96.10% C: 100%
Memory for Parameters (MB)	0.175 MB	0.177 MB	0.185 MB	0.024 MB
Estimated RAM Usage (MB) During Training	~ 2–4 MB	~2–4 MB	~ 2–4 MB	~ 1–2 MB

Analysing the performance of the studied networks (see Table 5), it is observed that feedforward neural networks (FNN1, FNN2, FNN3) achieve high accuracy ranging from 94.28 to 99.19%, while the CNN 1D model achieves 98.12%. The execution efficiency of the CNN 1D model suggests its applicability in real-time applications. Overall, very high accuracy (98.12%) can be observed based on the diagonal elements of the confusion matrix. The most significant misclassifications occur in classes L3 and W4 — L3 is misclassified as P2 (1 case) and P3 (3 cases), while W4 is confused with W3 (3 cases). The F1-score for most classes exceeds 96%, and for class C it reaches as much as 100%. The CNN1D model demonstrates a strong balance between classification performance and computational efficiency. Precision reached 100% for classes L and C. Resource consumption (see Table 5) is low across all models, with memory usage for parameters ranging from 0.024 MB to 0.185 MB, indicating lightweight architectures.

The FNN1 and FNN2 models achieved very similar and high values of Precision, Recall, and F1-score, exceeding 91% across all classes, indicating stable and effective classification performance. In comparison, the FNN3 model showed greater variability between classes, particularly for class *C*, where the F1-score dropped to 84.21%, suggesting difficulties in recognizing this category.

Detailed information on the computational efficiency of the models has been added Table 5 presents the training time of the models ranging from 5 577 ms to 8 469 ms depending on the architecture and the inference time per sample ranging from 3 ms to 5 ms The model size ranges from 24.55 KB for CNN1D to 176.97 KB for FNN2 and the number of parameters varies from 6 385 for CNN1D to 46 315 for FNN3 The estimated RAM usage during training is between 1 MB and 5 MB Measurements were performed on an Intel Core i7-8550U processor with a clock speed of 1.80 GHz and 8 logical threads allowing for a realistic assessment of computational requirements in resource-constrained environments The additional information enhances the practical value of the analysis and highlights the low computational demands of the models.

Regarding the proposed FNNs, these hold significant potential for solving general subtypes of HAR problems. Their short training time and high accuracy make them particularly attractive in resource-constrained environments, such as mobile devices. While they may not capture temporal dependencies as effectively as LSTMs, FNNs can still achieve satisfactory results in scenarios with stable activity patterns. Moreover, they offer significant flexibility in managing sequential dependencies between input data and outcomes, which can be particularly effective when integrated with recurrent or attention mechanisms in HAR tasks. Recurrent mechanisms, often implemented via LSTM networks, enable the model to retain information from previous time steps, which is crucial in HAR, where the temporal context of activities is vital for accurate classification. The question of whether it is better to recognize activities directly or through phase recognition is critical in HAR research. Direct recognition simplifies the classification process but may encounter difficulties with similar activities. In contrast, phase recognition offers greater accuracy as it allows the model to identify and classify individual segments before aggregating them into a complete activity. This approach can enhance the model’s resilience to variations in execution time or interruptions.

Regarding the CNN 1D as an FNN alternative, its automatic low- and high-level feature extraction ability reducing the need for extensive feature engineering is a significant advantage in HAR, where the complexity and variability of human activities can complicate manual feature extraction. Furthermore, CNNs are generally less susceptible to issues such as vanishing gradients compared to traditional recurrent networks, facilitating more stable training processes. However, CNNs typically require larger datasets to effectively mitigate overfitting, leading to increased computational complexity, extended training times, and higher resource requirements, which could limit its applicability in resource-constrained environments. In our research, the CNN model performed well, but a potential pitfall in its performance is the variability in accuracy depending on different activities, indicating challenges in distinguishing overlapping features.

In summary, the proposed offline model utilizing FNN and CNN architectures demonstrates strong capabilities in human activity classification, particularly in stable environments. The findings from the comparison with LSTM-AutoEncoder models^11^ suggest that while more complex architectures may offer better adaptability, simpler FNNs possess significant potential for efficient HAR applications.

## Conclusions and future work

The main conclusions of this work can be summarized as follows:

i. It was demonstrated that FNNs can satisfactorily classify complex human activities consisting of different phases as well as single-phase activities, even without paying particular attention to architecture optimization, provided that enough hidden layers and hidden neurons, i.e. enough trainable model parameters, are employed.
ii. Neural network inputs in the form of a variety of signals typically stemming from mobile phones rather than images or other high-dimensional inputs proved suitable for HAR.
iii. To achieve comparable efficacy with CNNs in HAR tasks, FNNs must employ innovative modeling of activities and preparation of the corresponding input data, ensuring that the learned patterns align with the task requirements and available computational resources.
iv. The temporal element that is an inherent necessity In HAR tasks, was successfully captured in the 80 point task description window.
v. The fixed 80 point temporal window makes FNNs a viable approach amenable to online HAR.

The main constraint is that all activities to be recognized should be fully represented by a series of 80 points applicable to all pertinent signals. In the general case, the number of points can be determined based on the product of the activity duration and sampling rate.

In conclusion, while CNNs are generally favoured in HAR for their ability to discern local patterns within temporal sequences, our findings suggest that FNNs remain competitive in specific HAR scenarios, achieving high accuracy and efficiency despite limitations in temporal feature extraction, particularly in the context of limited computational resources.

Future research could explore hybrid network architectures that effectively combine the advantages of both FNNs and CNNs, potentially leading to improved performance across a variety of HAR applications. Such hybrid approaches could integrate the sequential advantages of recurrent mechanisms with the feature extraction capabilities of CNNs, offering an improved comprehensive solution for classification performance of complex human activities.

## Data Availability

The data supporting the findings of this study are available from the corresponding author, T.N.R., upon reasonable request.

## Declarations

### Guideline statement

All methods were carried out in accordance with relevant guidelines and regulations.
